# Monoclonal Antibodies as Probes to Study Ligand-Induced Conformations of Troponin Subunits

**DOI:** 10.3389/fphys.2022.828144

**Published:** 2022-03-25

**Authors:** Monica Rasmussen, Jian-Ping Jin

**Affiliations:** ^1^Department of Physiology, Wayne State University School of Medicine, Detroit, MI, United States; ^2^Department of Physiology and Biophysics, University of Illinois at Chicago, Chicago, IL, United States

**Keywords:** protein conformation, ELISA, LSPR, monoclonal antibody, high-throughput screening, troponin, ligand-protein binding

## Abstract

Striated muscle contraction and relaxation is regulated by Ca^2+^ at the myofilament level *via* conformational modulations of the troponin complex. To understand the structure–function relationship of troponin in normal muscle and in myopathies, it is necessary to study the functional effects of troponin isoforms and mutations at the level of allosteric conformations of troponin subunits. Traditional methodologies assessing such conformational studies are laborious and require significant amounts of purified protein, while many current methodologies require non-physiological conditions or labeling of the protein, which may affect their physiological conformation and function. To address these issues, we developed a novel approach using site-specific monoclonal antibodies (mAb) as molecular probes to detect and monitor conformational changes of proteins. Here, we present examples for its application in studies of two subunits of troponin: the Ca^2+^-binding subunit, TnC, and the tropomyosin-binding/thin filament-anchoring subunit, TnT. Studies using a high-throughput microplate assay are compared with that using localized surface plasmon resonance (LSPR) to demonstrate the effectiveness of using mAb probes to assess ligand-induced conformations of troponin subunits in physiological conditions. The assays utilize relatively small amounts of protein and are free of protein modification, which may bias results. Detailed methodologies using various monoclonal antibodies (mAbs) are discussed with considerations for the optimization of assay conditions and the broader application in studies of other proteins as well as in screening of therapeutic reagents that bind a specific target site with conformational and functional effects.

## Introduction

The troponin complex plays a crucial role in regulating striated muscle contraction and relaxation by allosterically modulating the configuration of tropomyosin (TM) on the actin thin filament, thus regulating myosin cross-bridge cycling (Gordon et al., 2000). Troponin is a heterotrimer consisting of troponin C (TnC, Ca^2+^-binding subunit), troponin I (TnI, actomyosin ATPase inhibitory subunit), and troponin T (TnT, TM-binding subunit). Muscle contraction starts with initial conformational changes in TnC upon Ca^2+^ binding, which confers further conformational changes in TnI and TnT to release the inhibition of myosin ATPase and permit the movement of TM for cross-bridge formation (Zhang et al., 2011). Owing to the essential nature of the allosteric functions of troponin subunits in muscle contraction and relaxation, understanding their structure-conformation relationship and the conformational states relevant to muscle contraction and relaxation is vital to understanding how striated muscle functions.

Two homologous genes encode muscle-fiber type specific TnC isoforms, one expressed in fast skeletal muscle and the other in cardiac and slow skeletal muscles. Both isoforms contain four E-F hand divalent metal ion binding sites, two high affinity sites in the C-terminal domain that competitively bind both Ca^2+^ and Mg^2+^, and two low affinity sites in the N-terminal domain that favor binding of Ca^2+^ (Robertson et al., 1981). The two TnC isoforms differ in that all ion-binding sites are active in fast skeletal muscle TnC, while one of the N-terminal sites (Site 1) in cardiac/slow skeletal muscle TnC is inactive (van Eerd and Takahashi, 1975; Putkey et al., 1991). In both isoforms, the N and C domains are connected by a linker region which forms an alpha helical arrangement in fast skeletal TnC but is less well-resolved in the X-ray crystallography structure of cardiac/slow skeletal muscle TnC, suggesting this region may be highly flexible in the latter isoform (Vinogradova et al., 2005). In muscle cells, the C domain metal-binding sites are constantly occupied by Mg^2+^, whereas the rising cytosolic Ca^2+^ ions during muscle activation preferentially bind the N domain of TnC to initiate the conformational changes that confer contraction (Grabarek, 2011). Experimental data suggest that Mg^2+^ may be able to compete to some degree with Ca^2+^ for binding to the N domain of TnC though it cannot initiate conformational changes (Potter and Gergely, 1975; Leavis and Kraft, 1978; Rayani et al., 2021).

Three TnT isoforms exist in fast skeletal, slow skeletal, and cardiac muscles, respectively (Wei and Jin, 2016). The N-terminal domain of TnT is a hypervariable region with a highly diverged structure among isoforms and undergoes alternative splicing during muscle development (Jin et al., 2008). The N-terminal structural variations have been shown to modulate the conformation and function of the middle and C-terminal conserved regions of TnT (Biesiadecki and Jin, 2002; Zhang et al., 2006). An intriguing feature of the N-terminal variable region of avian fast skeletal muscle TnT is an alternatively spliced Glu and His rich segment uniquely expressed in adult pectoral muscles of the avian orders of *Galliformes* (Jin and Smillie, 1994; Ogut and Jin, 1998). This N-terminal variable segment of *Galliformes* fast skeletal muscle TnT contains repeating [H(E/A)EAH] motifs designated the Tx segment (Smillie et al., 1988), which has been shown to bind transition metal ions (Jin and Smillie, 1994). While this unique feature is not present in muscles of other species and is therefore not required for the basic function of troponin T, research from our lab has previously demonstrated that binding of Zn^2+^ to the N-terminal domain of chicken fast skeletal muscle TnT alters the local and global molecular conformations with functional impacts (Ogut and Jin, 1996; Wang and Jin, 1998).

Traditional approaches to study folded protein structure include methods such as X-ray crystallography, nuclear magnetic resonance spectroscopy, circular dichroism, and Förster resonance energy transfer (FRET). These methods often demand large amounts of purified protein at high concentration and highly specialized equipment, or they require external labeling of the protein, which can interfere with the very native structure seeking to be investigated (Alberts et al., 2002; Liu and Hsu, 2005; Raicu and Singh, 2013). The now widespread availability of computational models has expanded the ability to model protein structure *in silico*, but such methods still rely on experimentally determined conformation data and the need for empirical validation through biochemical and biophysical studies (Bhasin and Raghava, 2006). Therefore, there remains a need to assess native protein conformation and physiological structure–function relationship through assays that can be readily performed in the laboratory of individual investigators.

**Figure 1:**
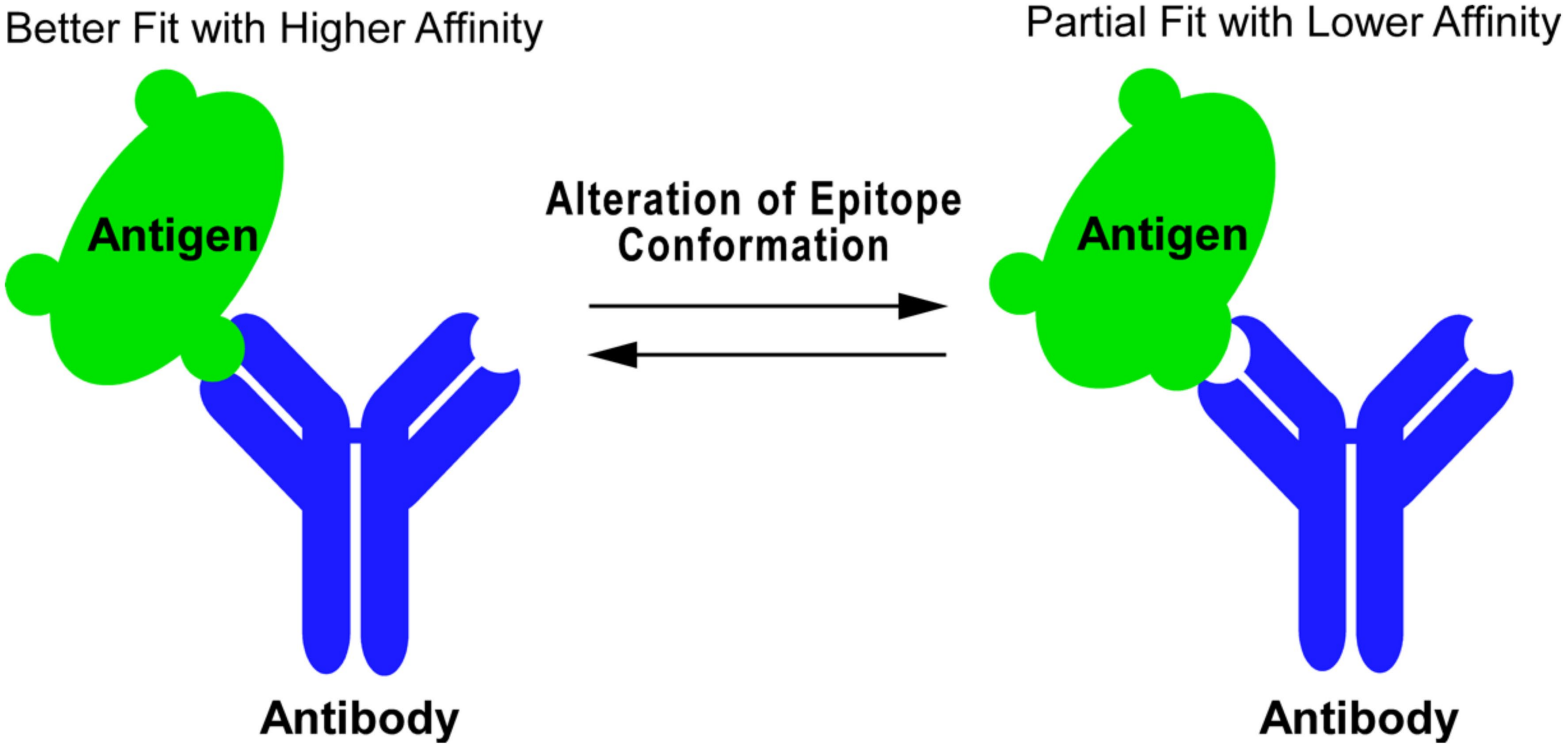
Antibody as probe to detect protein conformational change. Antibody–antigen binding is a protein–protein interaction whose affinity is determined by 3-D structural fitting of the antigenic epitope and the variable region of antibody. Changes in the epitope conformation can be reflected in the antibody binding affinity, where an alteration of the epitope conformation can shift an antibody–antigen interaction from a better fit with higher affinity (left) to a partial fit with lower affinity (right) and vice versa.

Antibodies are ubiquitously used in research laboratories. The binding of an antibody (immunoglobulin) to an antigen protein is a protein–protein interaction dependent on the 3-D conformational fit between the topology of the epitope and the variable region of the antibody. The affinity of an antibody to an antigen reflects the degree of structural fit (Figure 1). Generation of antibodies *in vivo* is mediated by antigen presenting cells, which process an immunogen and present it in the form of short peptides to activate B cells (Avalos and Ploegh, 2014). Therefore, antibodies generated by various forms of immunogens, such intact proteins, either denatured or native, as well as protein fragments or synthetic peptides can potentially be useful as conformational probes as long as their epitopes are related to the conformation-sensitive structures of a desired protein. Hybridoma technology has expanded the ability to produce site-specific monoclonal antibodies (mAbs) (Köhler and Milstein, 1975), allowing for the production of reagents which recognize various antigenic epitopes with high specificity.

**Figure 2:**
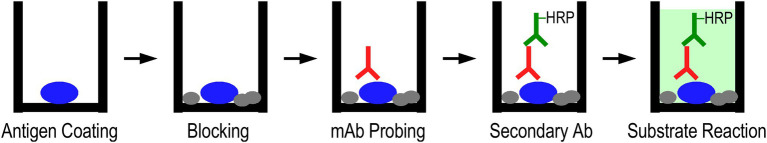
ELISA-based mAb epitope analysis. Protein of interest was coated on a microtiter plate in a buffer containing the desired additives, which can be used as the buffer throughout the entire assay or modified in later steps. After blocking the plate, the mAb conformational probe was incubated in serial dilutions, followed by washes and incubation with horse radish peroxidase (HRP)-conjugated secondary antibody. After final washes, the binding of mAb probes was detected *via* H_2_O_2_-ABTS colorimetric substrate reaction and recording the development of absorbance at 420 nm.

It is widely recognized that the high throughput enzyme-linked immunosorbant assay (ELISA) (Plested et al., 2003), typically performed in a 96-well microtiter plate, can rapidly titrate and quantitatively compare antibody-protein bindings. The ELISA method is easily adaptable to a wide array of study designs, among which we have explored its novel application in mAb protein conformational studies (Figure 2; Wang and Jin, 1998; Jin and Root, 2000; Chong and Jin, 2009).

**Figure 3:**
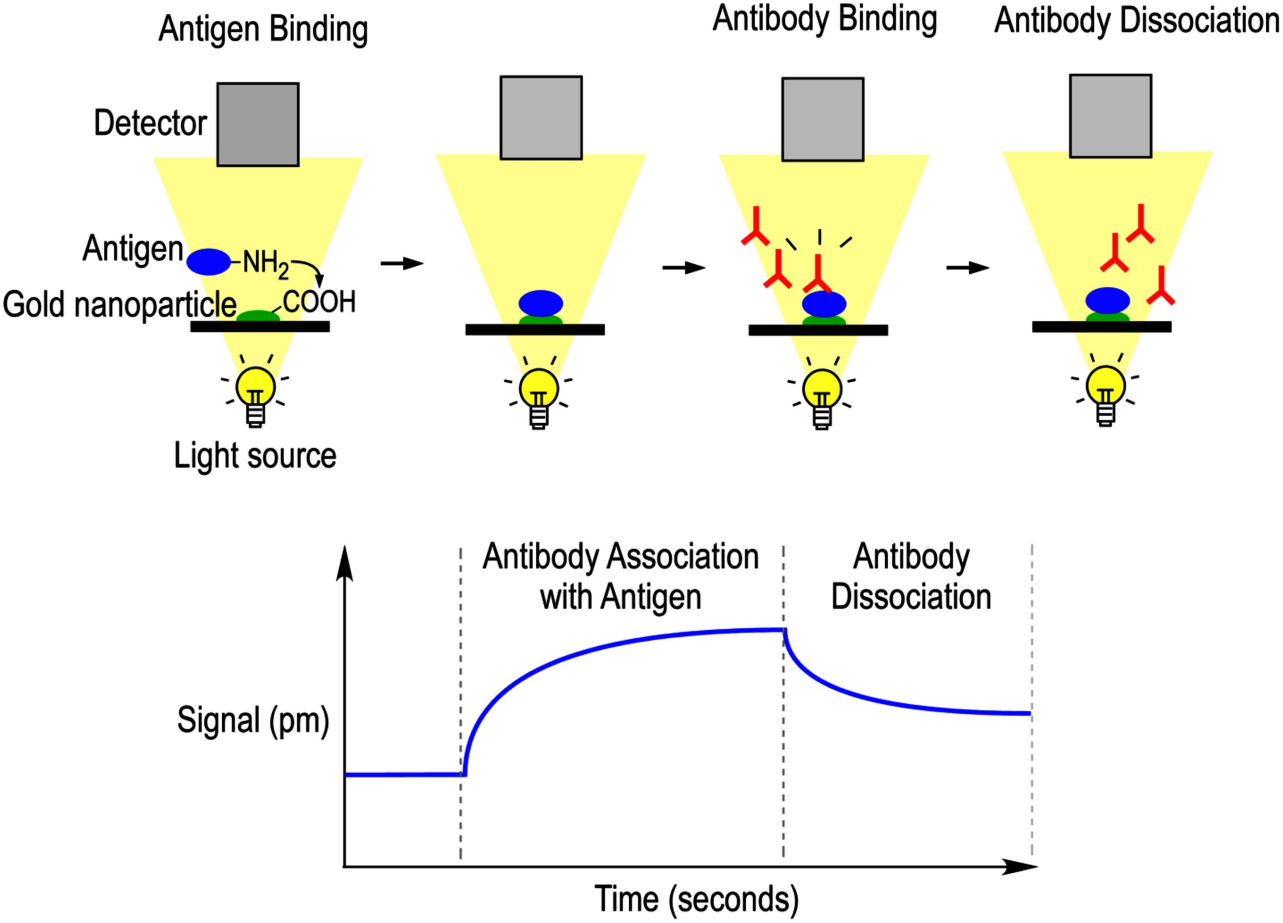
Localized surface plasmon resonance-based mAb epitope analysis. Protein of interest was attached to a carboxy-functionalized gold nanoparticle *via* covalent bonding with lysine residues. The protein was permitted to equilibrate in running buffer, and then the mAb probe diluted in the same buffer was permitted to flow over the chip and interacts with the protein. Interactions were measured *via* local changes in refractive index that are reflected in optical signal detection. Running buffer was then flushed over the chip to promote mAb dissociation. The resulting signal measured by the optical detector is plotted against time to calculate antibody association and dissociation rate constants.

Localized surface plasmon resonance (LSPR) is a technique which takes advantage of the oscillations of conduction band electrons in a metallic nanoparticle following stimulation by irradiated light. When probed with a light wave, the electron cloud will oscillate in a predictable manner, which will further shift with local changes at the surface of the nanoparticle. By functionalizing the nanoparticle, various proteins can be bound to the surface, and interactions with other proteins will disrupt the resonant wavelength in a quantitative manner (Petryayeva and Krull, 2011). This method can analyze a bound protein of interest using specific mAbs, in which empirical antibody–antigen binding data can be fit to a model to estimate association (K_a_) and dissociation (K_d_) constants. By using LSPR in conjunction with a high-throughput method like ELISA, a fuller picture of antibody–antigen binding can be efficiently studied (Figure 3).

In the present work, we employed a diverse range of site-specific mAbs to assess conformations of TnC and TnT for functional significance, with a focus on quantifying ligand-induced changes upon ion binding. We investigated Ca^2+^-induced changes in the molecular conformation of TnC at regions related to the E-F hand domains, as well as remote ligand-induced effects. We also explored how binding of transition metal ions to the N-terminal Tx segment affect local and overall conformation of avian pectoral muscle TnT. Site-specific mAbs are employed based on their epitope locations in TnC or TnT. LSPR is utilized to understand changes in TnC structure upon Ca^2+^ binding. We further discussed the utility of site-specific mAbs to investigate protein conformation, with an emphasis on optimization of assay conditions and the applicability of mAb analysis of protein conformational differences and changes for broader use in other systems, including the screening of therapeutic reagents that bind a specific target site with conformational and functional effects.

## Materials and Methods

### Proteins Used in Study

Recombinant chicken fast skeletal TnC and mouse cardiac/slow skeletal muscle TnC were expressed in BL21(DE3)pLysS *Escherichia coli* and purified as previously reported (Jin et al., 2007). Recombinant chicken fast skeletal muscle TnT8e16 protein was expressed in BL21(DE3)pLysS *E. coli* and purified as previously reported (Ogut and Jin, 1998). Recombinant chicken fast skeletal muscle TnT1 fragment 1–165 (N165) was expressed in BL21(DE3)pLysS *E. coli* and purified as previously reported (Ogut and Jin, 1996).

Concentrations of protein stocks were determined using Bradford reagent (BioRad) with a linear fit standard curve of serial dilutions of bovine serum albumin (BSA). Absorbances were determined at A_595 nm_ using an automated microplate reader (BioTek Synergy H1).

### Monoclonal Antibodies Used in Study

Development and immunological characterization of anti-TnC and anti-TnT mAbs were completed using the methods reported previously (Wang and Jin, 1998). Briefly, Balb/c mice were immunized with purified antigen using a short term protocol including a primary injection with Freund’s complete adjuvant and three daily pre-fusion boosts without adjuvant. Two days after the final boost, spleens cells were harvested and fused with SP2/0 mouse myeloma cells. Hybridoma clones were screened using ELISA against the antigen protein immobilized on 96-well microplate. The antibody-secreting hybridomas were subcloned three or more times to establish stable cell lines for use in the production of mAb-enriched mouse ascites fluid.

Anti-chicken fast skeletal muscle TnC mAbs 4E7, 2C3, and 2D10 were prepared as reported previously, through immunization with purified chicken fast skeletal muscle TnC (Jin et al., 2007). Anti-chicken fast skeletal muscle TnT mAbs 6B8, 3E4, 3H12, and 4C3 were prepared as described previously through immunization with purified chicken breast muscle TnT or leg muscle TnT (Wang and Jin, 1998). An anti-Tx peptide mAb 3C11 was produced as reported previously through immunization with a Tx3 peptide fused to ApoE protein with Freund’s complete adjuvant (Liu, 2016).

To partially purify mAbs from mouse ascites fluid, saturated ammonium sulfate solution was added dropwise to 1 ml of ascites fluid to arrive at 30% saturation. The mixture was permitted to equilibrate at 4°C with mixing for 1 h and then was centrifuged at 20,000 × *g* at 4°C for 15 min. The supernatant was collected, and the volume was measured. Saturated ammonium sulfate solution was added dropwise to the 30% saturation supernatant to arrive at 60% saturation, equilibrated at 4°C for 1 h, and centrifuged at 20,000 × *g* at 4°C for 15 min. The resulting pellet containing enriched IgG was collected and resuspended in phosphate buffered saline (PBS) for use.

### SDS-PAGE and Western Blotting

TnC and TnT proteins were analyzed *via* SDS-PAGE and Western blotting to determine the recognition by specific mAbs. Briefly, purified proteins in SDS-PAGE sample buffer were resolved on 14% Laemmli gel with an acrylamide-to-bisacrylamide ratio of 180:1 and were visualized using Coomassie blue R250 staining or electrically transferred to nitrocellulose membrane using a semi-dry apparatus (Bio-Rad). The membranes were blocked with Tris-buffered saline (TBS) containing 1% BSA, followed by incubation in TBS containing 0.05% Tween-20 (TBS-T), 0.1% BSA, and pre-titrated concentrations of mAbs at 4°C overnight. Membranes were washed using TBS containing 0.5% Triton X-100 and 0.1% SDS, and incubated with alkaline phosphatase-conjugated anti-mouse IgG secondary antibody (Santa Cruz) in TBS-T + 0.1% BSA at room temperature for 1 h. Membranes were washed again and developed in 5-bromo-4-chloro-3-indolyl-phosphate/nitro blue tetrazolium (BCIP/NBT) to visualize the target protein bands.

### Microtiter Plate Antibody Epitope Analysis of TnC and TnT Conformational Changes

As outlined in Figure 2, antibody epitope analysis using solid-phase ELISA was performed to assess changes in protein conformations *via* titration of antibody-protein binding affinities. To study the troponin subunits, a standard buffer mimicking the intracellular ionic environment (10 mM Tris–HCl, pH 8.0, and 100 mM KCl) was used with assay-specific additives described below. All assays were run in 96-flat bottom well microplates in triplicate.

To study Ca^2+^/Mg^2+^-dependent conformations of fast TnC, purified chicken fast skeletal muscle TnC was coated on microtiter plates at 2 μg/ml, 100 μl per well, in the standard buffer plus either 0.1 mM ethylene glycol tetraacetic acid (EGTA), 0.1 mM CaCl_2_, 3 mM MgCl_2_ + 0.1 mM EGTA, or 3 mM MgCl_2_ + 0.1 mM CaCl_2_, at 4°C overnight. Plates were washed three times 5 min each using the standard buffer with the corresponding assay-specific additives plus 0.05% Tween-20 to remove unbound protein, and the remaining plastic surface was blocked using 1% BSA in the washing buffer for 30 min. The immobilized protein was then incubated with serial dilutions of ascites fluid of anti-fast TnC mAbs 2C3, 4E7, or 2D10 in standard buffer plus 0.1% BSA, 0.05% Tween-20, and the assay-specific additives at 100 μl per well at room temperature for 2 h. Same as above, the plates were washed and further incubated with horse radish peroxidase (HRP)-conjugated anti-mouse secondary antibody (Santa Cruz) at room temperature for 1 h. Washed again to remove unbound secondary antibody, H_2_O_2_–2,2′-azinobis (3-ethylbenzthiazolinesulfonic acid; ABTS) substrate was added at 100 μl per well for color development at room temperature for 30 min. A_420 nm_ of each assay well was measured at a series of time points using an automated microplate reader (BioTek Synergy H1). Values in the linear range of color development were used to construct affinity titration curves after background subtraction.

To study the effects of transition metal binding to the N-terminal Tx element of avian pectoral muscle fast TnT on local and global molecular conformation, purified chicken fast TnT8e16 was coated at 4 μg/ml in the standard buffer containing 0.1 mM ethylenediaminetetraacetic acid (EDTA) or 0.1 mM ZnCl_2_. Microplate epitope analyses were performed as above using mAbs 3C11, 3E4, 4C3, 2C8, or 3H12.

### Localized Surface Plasmon Resonance Epitope Analysis of Antibody Kinetics

As illustrated in Figure 3, LSPR was employed to study TnC epitope conformation *via* determining the binding kinetic rate constants of mAbs. Chicken fast skeletal muscle TnC was covalently immobilized on a carboxyl sensor chip of the Open SPR™ instrument (Nicoya Lifesciences, Canada), using 1-ethyl-3-(3-dimethylaminopropyl)carbodiimide (EDC) and N-hydroxysuccinimide (NHS) to activate the carboxyl groups by producing an activated NHS-ester intermediate, which was then displaced by primary amines from lysine groups of the protein of interest. A 200 μl injection of fast TnC at 50 μg/ml in sodium acetate buffer pH 5 was allowed to bind to the chip over 5 min at a flow rate of 20 μl/min, followed by a blocking solution (Nicoya Lifesciences, Canada) to block any remaining activated esters on the chip.

To study antibody binding to the immobilized protein, ammonium sulfate-enriched mAb stock was diluted in the running buffer at 50 and 10 μg/ml to a negligible amount of ammonium sulfate. The mAb was permitted to bind to the immobilized protein over 300 s at a flow rate of 20 μl/min, followed by fresh running buffer for 180 s at 20 μl/min to allow for dissociation. The chip was regenerated with 3 M KCl (or HCl, pH 1.5, if salt was not sufficient to promote full regeneration). If a buffer change was required, the buffer was perfused through the instrument at 20 μl/min for 10 min for complete solution exchange. The data were retrieved and analyzed using TraceDrawer software, and the curves were fit to a one-to-one model to estimate binding constants (Myszka et al., 1997; Önell and Andersson, 2005; Rich and Myszka, 2010).

The effect of Ca^2+^ on TnC conformation was studied in buffers at pCa 10.0, 9.0, 8.0, 7.5, 7.0, 6.5, 6.3, 6.0, 5.5, 5.0, 4.5, and 4.0 prepared by mixing pCa 10.0 and 4.0 buffers [10 mM piperazine-N,N′-bis(2-ethanesulfonic acid) pH 7, 100 mM KCl, 3 mM MgCl_2_, 10 mM EGTA, 3 μM, or 10.06 mM CaCl_2_] calculated using the UC Davis Ca-Mg-ATP-EGTA v1.0 computer program^1^ and mixed at various ratios as described previously (Feng and Jin, 2020).

## Results

### Conformational Effects of Ca^2+^ and/or Mg^2+^ Binding to N and C Domains of Fast Skeletal Muscle TnC Detected Using mAb ELISA

**Figure 4:**
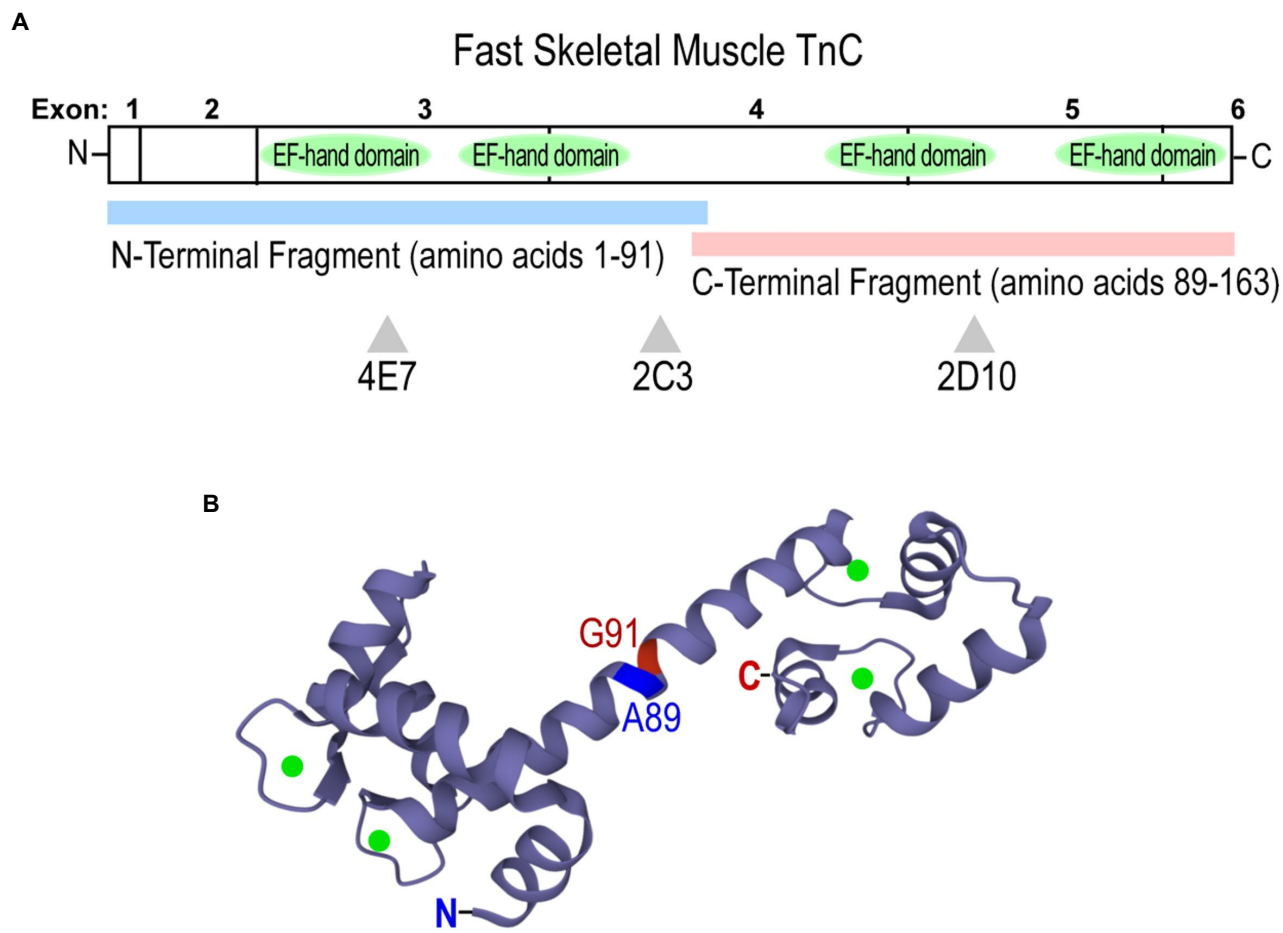
Structure of chicken (*Gallus gallus*) fast skeletal muscle TnC. **(A)** A linear map showing locations of epitopes recognized by anti-TnC mAbs 4E7, 2C3, and 2D10 used as conformational probes in the present study. Fast TnC consists of N and C terminal domains containing four E-F hand Ca^2+^-binding motifs, along with a central helical linker to connect the domains. N-terminal (amino acids 1–91) and C-terminal (89–163) fragments were employed to map epitopes of the mAbs. **(B)** A crystal structure of fast TnC in the Ca^2+^-saturated state (PDB: 1YTZ; Ca^2+^ ions are present as green spheres). The N and C termini, and the boundary of the N- and C-terminal fragments used for mAb epitope mapping are indicated.

Domain-specific anti-chicken fast skeletal muscle TnC mAbs 4E7, 2C3, and 2D10 were employed to assess conformational changes of fast TnC induced by the binding of Ca^2+^ and Mg^2+^. The epitope locations of the mAbs have been previously reported (Jin et al., 2007), with 4E7 and 2C3 against the N domain fragment (amino acids 1–91) and 2D10 against the C domain fragment (amino acids 89–163; Figure 4A). Chicken fast skeletal TnC contains 4 E-F hand metal-binding sites: the high-affinity C domain sites bind Mg^2+^ or Ca^2+^, while the low-affinity N domain sites preferentially bind Ca^2+^ (Figure 4B; Vinogradova et al., 2005; Sehnal et al., 2021). The three mAbs were used as probes in an ELISA microtiter plate solid-phase assay to assess changes in fast TnC conformation with Ca^2+^ and/or Mg^2+^ binding and with an EGTA control for the metal-free apo form.

**Figure 5:**
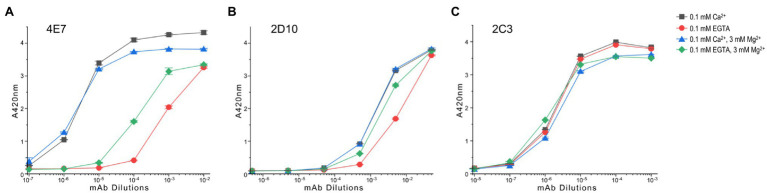
Conformational effect of Ca^2+^ and/or Mg^2+^ binding to fast TnC detected in microtiter plate ELISA. **(A)** The ELISA titration curves show that mAb 4E7 against the N domain of fast TnC exhibits a low affinity in the metal-free condition. The presence of Mg^2+^, which binds to the C domain sites, significantly increased the affinity of mAb 4E7, indicating an effect on the N-domain conformation. Binding of Ca^2+^ to the N-domain sites in the presence of 3 mM Mg^2+^ further increased the affinity of mAb 4E7. The highest affinity of mAb 4E7 was seen in the four-Ca^2+^ state, indicating that the occupations of the C domain sites by Ca^2+^ or Mg^2+^ have different conformational effect on fast TnC. **(B)** mAb 2D10 against the C domain of fast TnC shows higher affinities in the four-Ca^2+^ or Ca^2+^ + Mg^2+^ state than that of Mg^2+^ alone while the metal free-apo state has the lowest affinity, indicating both ions bind to the C-domain with similar conformational effect whereas Ca^2+^ binding to the N domain increases the affinity for mAb 2D10. **(C)** mAb 2C3 detected a conformation-independent epitope that displays similar affinity for fast TnC in the presence or absence of Ca^2+^ and Mg^2+^ ions.

The ELISA mAb titration curve in Figure 5A shows that fast TnC at the physiological activation state (two-Mg^2+^ and two-Ca^2+^) exhibits significantly higher affinity for 4E7 in comparison to that of the EGTA-apo state. Occupation of the C domain metal binding sites by Ca^2+^ further increases the affinity to mAb 4E7 (Figure 5A). The Mg^2+^-only relaxation state shows an intermediately higher binding affinity than that of apo state (Figure 5A). The results, therefore, indicate that the binding of Ca^2+^ to the N domain of TnC during muscle activation reconfigures the N domain conformation in a detectable manner *via* change of mAb 4E7 affinity. The effects of four-Ca^2+^ vs. Ca^2+^ + Mg^2+^ on increasing mAb 4E7 affinity further demonstrate a cross talk between the C and N domains.

The anti-C domain mAb 2D10 shows higher affinity than that of the metal free-apo state in the presence of either Ca^2+^ alone or Ca^2+^ + Mg^2+^ ions (Figure 5B), indicating structural effects regardless of which ion occupies the C domain binding sites. The detectable difference between the Mg^2+^ only state and the Ca^2+^ + Mg^2+^ state (Figure 5B) indicates a Ca^2+^-N domain binding-induced conformational change in the C domain.

In contrast, the affinity of mAb 2C3 was not dramatically affected by the binding of Ca^2+^ and Mg^2+^ as compared to the EGTA-apo state (Figure 5C). In addition to confirming consistent coating of TnC protein on the microtiter plate regardless of metal additives, the result suggests that the mAb 2C3 epitope may lie in the central helical linker region near the end of the N domain peptide of fast skeletal TnC (Figure 4A) and may be conformationally independent of the four metal ion-binding sites.

### Ca^2+^ Sensitivity of Fast Skeletal Muscle TnC Titrated With LSPR

**Figure 6:**
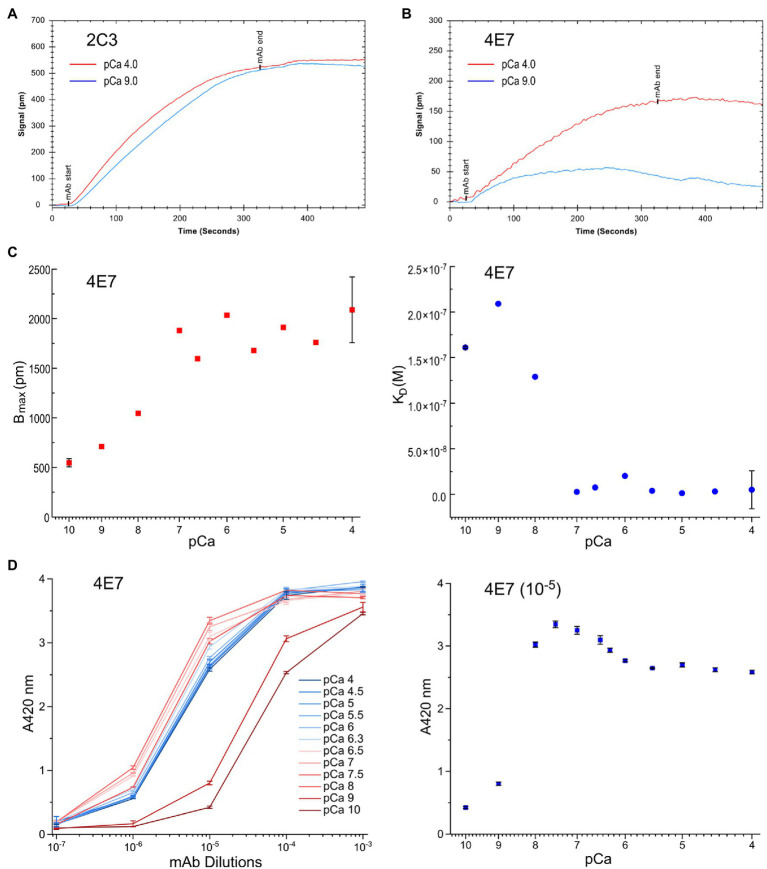
Conformational effect of Ca^2+^ binding to fast TnC detected in quantitative LSPR assay. **(A)** Changes in buffer pCa did not affect the binding of mAb 2C3, consistent with the observation in ELISA that mAb 2C3 binding to fast TnC remains constant regardless of ion binding. In addition, the binding signal of mAb 2C3 confirms constant protein coating on the LSPR chip. **(B)** In contrast, the binding of mAb 4E7 against the N domain of fast TnC changes with the change in pCa. A significant decrease in association rate and increase in dissociation rate at pCa 9.0 vs. pCa 4.0 are detected, indicating weaker binding of the mAb probe in the Ca^2+^-free state. **(C)** The physiological range pCa titration curve of mAb 4E7 binding with LSPR indicates that fast TnC changes conformation when [Ca^2+^] increases. The maximum binding of mAb 4E7 to the fast TnC chip, B_max_, increased in a gradual manner to a plateau level when [Ca^2+^] reached pCa 7.0, while K*_D_*, the equilibrium dissociation constant, started to decrease when [Ca^2+^] increases to pCa 8.0 and rapidly minimized at pCa 7.0. **(D)** The ELISA titration curves of mAb 4E7 binding to fast TnC at the same range of pCa (left panel) show a dramatic increase in affinity from pCa 10.0 to pCa 8.0. At sub-saturated concentration of mAb 4E7 (10^−5^ dilution, right panel), the [Ca^2+^] titration curve reveals three-phases of binding affinity changes: A rapid increase from pCa 10 to 7.5, a small decrease from pCa 7.5 to 5.5, and a plateau above pCa 5.5.

Using mAbs 2C3 and 4E7 in LSPR infused with buffers containing 3 mM Mg^2+^ and differing [Ca^2+^], mAb 2C3 affinity to fast skeletal muscle TnC is nearly identical at pCa 9.0 and 4.0 (Figure 6A), consistent with the results from the ELISA titrations. mAb 4E7, on the other hand, shows a low association rate with fast skeletal muscle TnC at pCa 9.0 with a dramatic increase at pCa 4.0 (Figure 6B). Infusing mAb-free buffer to promote dissociation quickly returns the pCa 9.0 curve to baseline, conditions which mimic the post-wash endpoint readings in ELISA.

Over the range from pCa 10.0 to 4.0, a shift in mAb 4E7 affinity is observed with [Ca^2+^] at and above pCa 8.0 (Figure 6C). The Ca^2+^-induced conformational changes at the mAb 4E7 epitope, reflected by the equilibrium dissociation constant *K_D_*, mirror the equilibrium endpoint measured in ELISA. While B_max_, the maximum possible binding to the fast TnC chip, increases with increasing [Ca^2+^], *K_D_* decreases, reflecting a higher affinity of mAb 4E7 for fast TnC in the Ca^2+^-bound state. In our previous work using skinned cardiac muscle fiber preparations with similar buffer conditions, pCa 5.8 is approximately the concentration of half maximal force activation, indicating a Ca^2+^ concentration sufficient to induce TnC conformational changes that instigate muscle contraction (Wong et al., 2019). Of note, both measures of antibody kinetics, B_max_ and K*_D_*, reach an equilibrium value at approximately pCa 7.0. The results implicate that fast TnC can be sufficiently “primed” at this pCa prior to the fiber activation of contraction at pCa 5.8, similar to the four-state model of calcium regulation of muscle contraction proposed in a previous study of cardiac muscle (Sun and Irving, 2010).

The results of an ELISA pCa titration of mAb 4E7 binding to fast TnC further validate the results observed in LSPR (Figure 6D), where 4E7 shows a dramatic increase in affinity from pCa 10 to 7.5 followed by a gradual decrease to reach a plateau level at pCa 5.5. With the presence of two Ca^2+^ binding sites in the N domain of fast TnC, we posit that the early phase of mAb 4E7 epitope conformational change from pCa 10 to 7.5 may be reflective of one vs. two Ca^2+^ state binding, whereas the second phase conformational change between pCa 7.5 and 5.5 induces myofilament activation and force production, though more work needs to be done to further validate this hypothesis.

### Conformational Effect of Transition Metal Ion Binding to Tx-Segment of Avian Pectoral Muscle TnT

**Figure 7:**
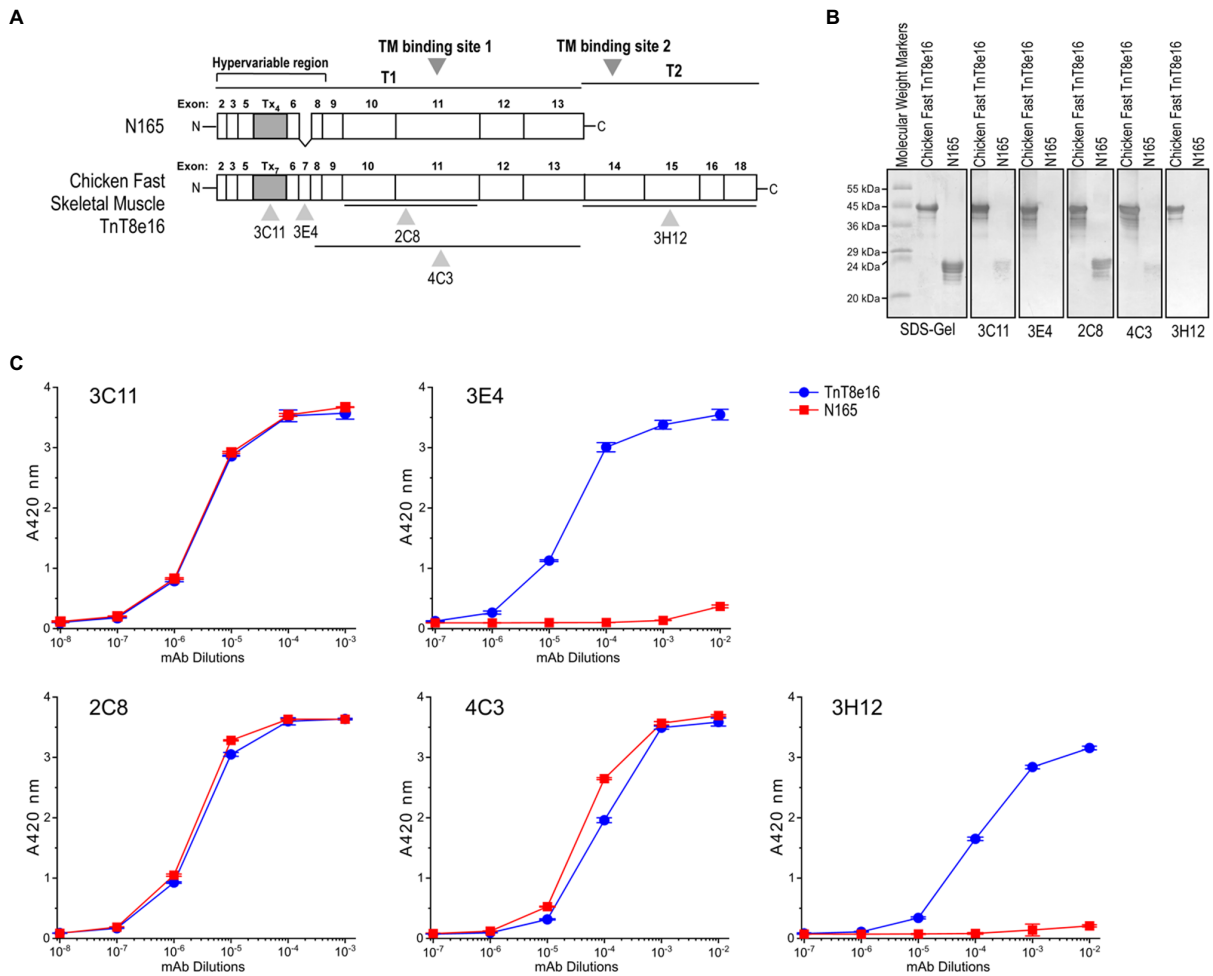
Linear structure of chicken fast skeletal muscle TnT8e16 and epitope mapping of anti-TnT mAbs. **(A)** Locations of the epitopes recognized by anti-TnT mAbs used in the present study are indicated on a linear map of chicken fast TnT8e16. The Tx segment of chicken fast TnT, consisting of seven repeated H(E/A)EAH transition metal binding motifs, is encoded by alternatively-spliced exons. **(B)** Western blotting and **(C)** ELISA using intact chicken TnT8e16 and N-terminal T1 fragment N165 demonstrated that mAbs 2C8, 4C3, and 3C11 epitopes are contained within the N165 peptide, while the 3H12 epitope is outside of N165. mAb 3E4 had no reaction with N165 due to the lack of exon 7-encoded segment, serving as a background control.

Site specific mAbs 3C11, 3E4, 2C8, 4C3, and 3H12 against the pectoral splice form of chicken fast skeletal muscle TnT were employed to assess conformational changes with metal ion binding. Previous characterizations have shown that mAb 3C11 is specific to the metal binding Tx peptide, mAb 3E4 is against an epitope lying in the adjacent exon 7-encoded linker region, and mAb 2C8 epitope lies downstream in the exons 10–11 encoded segment (Figure 7A; Wang and Jin, 1998; Jin and Chong, 2010; Liu, 2016). The Western blots and ELISA titrations against intact TnT8e16 and the T1 segment of chicken fast skeletal TnT1, N165, further determined the epitope locations of the anti-TnT mAbs used in the present study. mAb 2C8, used as a positive control, recognizes the N165 peptide and displays equal reactivity to N165 and TnT8e16 in both Western blot and ELISA (Figures 7B,C). mAbs 3C11 and 4C3 showed similar reactive pattern to TnT8e16 and N165 in ELISA and thus also possess epitopes within the T1 segment. N165 lacks the exon 7-encoded segment, and thus mAb 3E4 displays reactivity to TnT8e16 but not to N165. Both mAbs 3C11 and 4C3 lacked strong reactivity to N165 in Western blot, suggesting low affinity under the SDS-PAGE denaturing conditions as compared to the native conformation observed in ELISA. mAb 3H12 does not react to N165 in Western blot or ELISA, indicating its epitope is outside of the T1 segment in the C-terminal T2 region.

**Figure 8:**
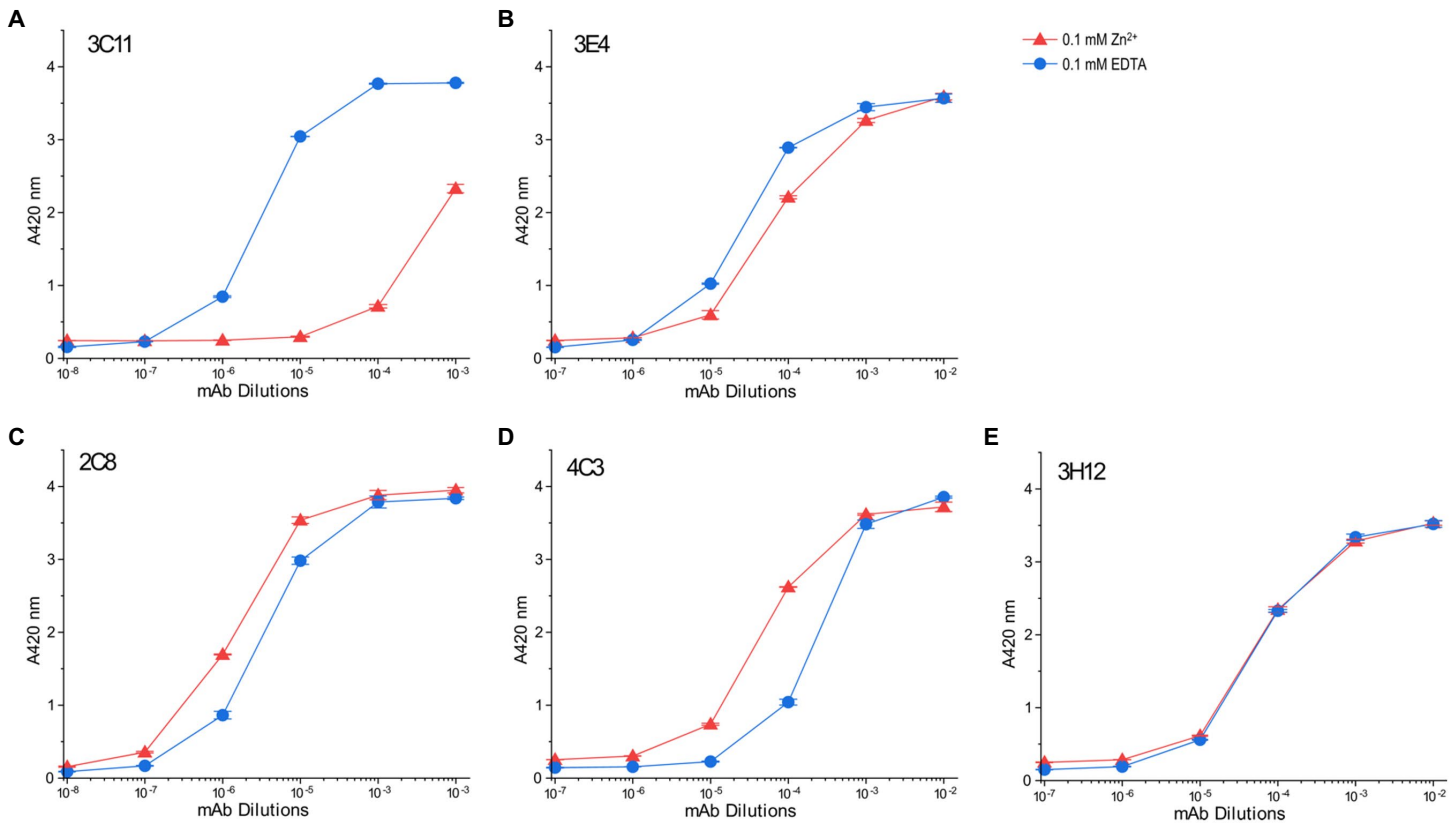
Conformational effect of metal ion binding to chicken fast skeletal muscle TnT8e16 detected by ELISA epitope analysis. **(A)** Zn^2+^-binding to the N-terminal Tx segment of TnT8e16 resulted in a dramatic decrease in the affinity of anti-Tx mAb 3C11 as compared to the ethylenediaminetetraacetic acid (EDTA) control. **(B)** mAb 3E4 raised against an epitope related the exon 7-encoded segment downstream of the Tx metal binding site also showed decreased affinity with the binding of Zn^2+^, though less dramatic, reflecting conformational effects propagated from the metal-Tx binding. **(C)** Zn^2+^ binding to the Tx segment enhanced the binding of mAb 2C8 against the exons 10–11 encoded segment further downstream in comparison to the EDTA control. **(D)** mAb 4C3 shows an effect similar to that of mAb 2C8. **(E)** mAb 3H12 against an epitope in the T2 domain of TnT8e16 remote from the Tx segment did not detect any Zn^2+^ binding-induced conformational change. In addition to reflecting that the T2 domain of TnT may conform largely independent of the N-terminal variable region, the result served as a protein coating control.

Binding affinities of those mAb probes to TnT8e16 were compared between the Zn^2+^-bound and EDTA-apo states to investigate the metal-Tx binding-induced conformational changes. Owing to the large number of site-specific mAbs available, we were able to map conformational changes directly at the Tx segment as well as highlight ligand-induced effects felt further from this domain. mAb 3C11 displayed a clear detection of the local conformational effect of Zn^2+^ binding on the Tx cluster, whereby which the affinity of mAb 3C11 was significantly decreased relative to the EDTA-apo control (Figure 8A), as previously observed in other anti-Tx mAbs (Wang and Jin, 1998). The binding of mAb 3E4 against a closely downstream epitope also displayed a decrease in affinity with Zn^2+^ binding, though the effect was less dramatic, indicating a propagated conformational change (Figure 8B).

Interestingly, mAbs 2C8 and 4C3 recognizing epitopes further downstream detected similarly increased affinities to TnT8e16 in metal-Tx-bound state than that to the EDTA-apo state (Figures 8C,D). This effect suggests the entire T1 segment may be highly flexible and responds with metal ion binding to change conformation and function.

The results also indicate that mAbs raised during immunizations may differentiate to have their variable regions fit with either metal-bound or metal-free state of TnT8e16. Since the antigenic epitopes are presented to the B lymphocytes in short peptides, and the 2C8 and 4C3 epitopes are remote from the N-terminal Tx segment (Figure 7A), the yield of mAbs with higher affinity for the metal bound state indicates that the metal-Tx binding induced conformational reconfiguration in the middle region of avian pectoral muscle TnT reflects an intrinsic property present in isolated antigenic peptides.

In contrast, the affinity of mAb 3H12 did not change with the binding of Zn^2+^ to the N-terminal Tx element (Figure 8E). The epitope of mAb 3H12 is contained within the C-terminal T2 segment, remote from the Tx region, implying that the C-terminal T2 domain conforms independently of the T1 domain and is insensitive to metal ion-Tx-binding induced conformational changes. The results of mAb 3H12 binding also confirm consistent protein coating regardless of buffer conditions, serving as a control and validating the ELISA system for use in the comparison of anti-TnT8e16 mAbs.

## Discussion

The methods and results described the present study highlight the utility of mAbs as site-specific probes to study protein conformation and changes. Our goal is to provide a framework for designing experiments, which measure changes both locally at the effector site as well as at remote allosteric regions. We employed two subunits of the troponin complex, TnC and TnT, to test the effects of metal ligand binding and demonstrate this novel approach in ELISA and LSPR. Several points on the methodology as well as findings on TnC and TnT structure–function relationships are summarized as follows.

### mAbs As Conformational Probes to Study Protein Structure–Function Relationships

The use of antibodies to measure epitopic structure and conformational changes of proteins has many advantages. Typically, only small amounts of protein are required for such assays, and no protein labeling is required, avoiding bias in the results. Furthermore, the results of our study illustrate how mAb-based structural epitope assays can be designed in both high throughput, semi-quantitative (ELISA), and kinetically quantitative (LSPR) manners. Of particular interest is whether protein structure and conformation, when perturbed, change in a gradual or quantum fashion. For example, the pattern of changing affinity of anti-TnC mAbs with changes in pCa shown in the present study may reflect how troponin conformation responds to the rising of intracellular Ca^2+^ during the activation of muscle contraction. Although the results may be reflective of an averaging effect at the surface of the microtiter plate or LSPR chip, whereby which at threshold pCa levels, some TnCs are Ca^2+^-bound while others are not, the condition is similar to that of TnC immobilized in myofilament and is therefore physiologically informative.

Crucial to the execution of the approaches is the availability of a range of mAbs raised against different portions of the protein of interest. With the advent of hybridoma technology, there is an ability to raise a large number of hybridoma clones in a single immunization and then screen for mAbs of various epitope specificity. Many commonly available mAbs against various proteins of interests can also be evaluated for use as conformational probes in the approaches described in the present study.

The immunogenicity of epitope structure of proteins may limit the ability to develop mAbs against certain parts of a protein, and there is a need to map the epitopes recognized by available mAbs in order to design an informative study. Still, once characterized, specific mAbs possess high utility and can be adapted to many study designs. To further optimize study design, one can take advantage of the high-throughput nature of ELISA to perform a broad screening of available mAbs, mapping epitopes, optimizing dilutions, and testing a large range of biochemical, physiological, and pathological conditions which may alter protein structure. LSPR can be of complementary use to provide a quantitative measurement of antibody affinity once study conditions have been determined, and the data in tandem give a holistic picture of a given protein’s structure and serve to independently validate results. Main features of the ELISA and LSPR approaches are summarized in Table 1.

**Table 1:** Comparison of advantages and disadvantages of ELISA vs. localized surface plasmon resonance (LSPR) technology.

ELISA	LSPR
Higher through-put using microplates	Lower through-put
Provides only endpoint monoclonal antibodies (mAb) binding	Provides estimation of mAb kinetic on- and off-rates
Buffer change possible in each step of assay	Buffer change possible in each step of assay
Assays run on multiple mAb populations in parallel	Assays run on single mAb population that is regenerated
No plate regeneration necessary	Background signal reduction and chip regeneration optimization crucial for quality data
Uses standard microplate readers and lower cost microtitering plates	Requires specialized instrument and higher cost LSPR chips
Useful for broad screening of multiple assay conditions and mAbs	Useful for quantitative and kinetic validation of single mAb-antigen interaction

### Ca^2+^ and Mg^2+^ Modulate Local and Remote Conformational Changes in TnC

TnC belongs to the calmodulin family of Ca^2+^-binding proteins, and the data obtained in the present study demonstrate how Ca^2+^ binding to the N domain of fast TnC induces conformational modulations which can be clearly displayed as changes in the affinity of mAb 4E7 (Figure 5A). LSPR data confirm the results of ELISA with quantitative antibody association and dissociation rates (Figure 6).

An interesting observation is that Mg^2+^ binding to the C domain sufficiently flexes the N domain to induce measurable changes (Figure 5A). On the other hand, Ca^2+^ binding to the N domain does not dramatically alter the molecular conformation of C domain, as shown by the relatively small change in the affinity of mAb 2D10 (Figure 5B). It is also worth noting that while the conformational cross talk between the N and C domains of fast TnC is anticipated to transmit *via* the central helical linker (Figure 4B), the helix *per se* seems to have no significant change in epitopic conformation as shown by the lack of change in mAb 2C3 affinity (Figure 5C).

Since it is not possible through our studies, both ELISA and LSPR, to distinguish the ligand-induced conformational changes of a single protein, we must consider our data to be the result of an averaging of the population of TnC’s bound and reflective of TnC-Ca^2+^ binding at equilibrium, whereby which at an intermediate pCa, some TnC’s may be ion-bound while others are not. In our work, we consider that TnC-Ca^2+^ binding during muscle fiber activation arising from the increase cytosolic Ca^2+^ models a similar situation, with muscle fiber activation resulting from the conformational change of multiple troponin repeats along the thin filament. Our observed feature of the three phase responses of fast TnC to rising [Ca^2+^] (Figure 6D) may reflect the contractile kinetics of myofilaments with physiological and pathological significance.

### The Unique Transition Metal Binding Cluster Tx in Avian Flight Muscles and Potentially Functional Role of Metal-Ion Induced Conformational Modulation

Adult pectoral muscle-specific fast skeletal muscle TnT in *Galliformes* birds, represented by chicken TnT8e16, contains a unique transition metal ion binding Tx segment that can be targeted to induce local and remote conformational changes in TnT. Using a battery of site-specific anti-TnT8e16 mAbs, we were able to investigate epitopic conformational changes at five different points (Figure 7A) for how metal ion-Tx binding alters the structure and function of TnT.

While the dramatically decreased affinity of the anti-Tx mAb 3C11 in 0.1 mM ZnCl_2_ (Figure 8A) confirms previous observation that metal binding significantly alters the local conformation of the Tx segment, mAb 3E4 against a nearby epitope (Figure 7A) detected a weaker secondary effect propagated from the Tx site (Figure 8B), demonstrating the conformational modulatory role of Tx-metal binding.

Monoclonal antibodies 2C8 and 4C3 against epitopes in the middle region of TnT (Figure 7A) both detected metal binding-induced conformational changes (Figures 8C,D). In contrast to the metal-binding induced decreases in the affinities of anti-Tx mAb 3C11 and mAb 3E4 against a nearby epitope, the binding of Zn^2+^ to the N-terminal Tx segment induced increased affinities for mAbs 2C8 and 4C3. This finding suggests that Zn^2+^ binding to the Tx segment likely increases the flexibility of the middle region of TnT, allowing increased compliance and fitting to the variable region of the mAb probes. The alternative splicing regulated Tx segment is specifically expressed adult avian flight muscles. The data may imply that the trace amount of Zn^2+^ in avian pectoral muscle cells may bind to Tx to produce a remote conformational effect on the function of the TM binding site 1 in the T1 region with functional importance, a hypothesis worth further investigation.

The anti-T2 region mAb 3H12 did not detect any conformational change (Figure 8E). Therefore, the N-terminal originated conformational modulations seem predominantly through changes in the T1 region (Figure 7A).

### High Throughput ELISA mAb Epitope Confirmational Analysis

Enzyme-linked immunosorbent assay is a high throughput, well-established methodology platform using microtiter plates of 96- or 384-well format of which the former is commonly used with standard plate readers. Even with manual operations, a trained operator can readily handle 4–6 plate in daily assays of several hundreds of samples or testing conditions. The steps of microtiter plate ELISA can be automated by using a plate washer and a multi-channel sample handler to maximize the high throughout benefit.

The ELISA mAb conformational analysis described in the present study provides a generally practical approach to conduct high throughput investigation on protein structure–function relationships and ligand-induced functional changes in various proteins. The assay can be done in physiological buffers to mimic *in vivo* conditions. One valuable feature of the microtiter plate assay is that the buffer and environmental conditions can be different for the protein coating, ligand binding, antibody incubation, and washing steps to compensate for the solubility of reagents and optimal temperature and pH of the reactions. Specific interactions identified using high throughput ELISA screening can be further investigated using more quantitative or high resolution but low throughput approaches for in depth characterization.

### Broader Applications

Protein conformation is directly related to function. The mAb epitope conformational analysis approach can be considered for various applications in addition to myofilament protein studies. For example, while the current work focused on ligand-induced changes in the ion-binding subunits of troponin, N-terminal phosphorylation of cardiac TnI is a well-documented phenomenon with functional effects (Solaro et al., 1976), and phospho-specific mAbs have been developed to assess phosphorylation states (Al-Hillawi et al., 1998). Previous work from our lab has shown that N-terminal phosphorylation or restrictive truncation induced conformational changes in a remote region of cardiac TnI are detectable *via* an mAb probe (Akhter et al., 2012). Future directions may focus on understanding how TnI conformation changes with phosphorylation and what role this plays in the inhibitory function of cardiac TnI.

Pathogenic mutations represent another avenue with implications for human health. Disease-causing mutations in a protein may not be directly at the functional site responsible for the disease phenotype but instead may act through remote or global conformational effects. Therefore, one can use mAb conformational analysis to detect such effect of pathogenic mutations of a protein and obtain insights into possible therapeutic intervention.

Comparing the mAb epitope analysis with other methods for conformational studies, as well as combining ELISA’s high throughput nature with LSPR kinetic analysis, another example for broader applications of the mAb epitope conformational analysis is drug screening. With a conformationally sensitive mAb against the target protein, ELISA can sensitively detect conformational changes induced by ligand binding. The sensitivity can be increased by using combinations of two or more mAbs against different epitopes or use a polyclonal antibody at proper dilution. Upon identification of promising compounds that induce conformational changes in the target protein, biochemical, cellular, and organ level functional studies can be conducted to further select candidates for therapeutic development.

## Data Availability Statement

The original contributions presented in the study are included in the article/supplementary material; further inquiries can be directed to the corresponding author.

## Author Contributions

MR designed and conducted the experiments, analyzed results, drafted the manuscript and figures, and edited and approved the submission. J-PJ conceived the research, designed the experiments, drafted the manuscript and figures, and edited and approved the submission. All authors contributed to the article and approved the submitted version.

## Funding

This study was supported in part by grants from the National Institutes of Health (HL127691 and HL138007 to J-PJ).

## Conflict of Interest

The authors declare that the research was conducted in the absence of any commercial or financial relationships that could be construed as a potential conflict of interest.

## Publisher’s Note

All claims expressed in this article are solely those of the authors and do not necessarily represent those of their affiliated organizations, or those of the publisher, the editors and the reviewers. Any product that may be evaluated in this article, or claim that may be made by its manufacturer, is not guaranteed or endorsed by the publisher.

## Footnotes

^1^ https://somapp.ucdmc.ucdavis.edu/pharmacology/bers/maxchelator/CaMgATPEGTA-NIST.htm
